# Sequential intravesical Bacillus Calmette-Güerin and mitomycin C applied with electromotive drug administration therapy for non-muscle invasive bladder cancer

**DOI:** 10.3389/fonc.2025.1682918

**Published:** 2025-10-08

**Authors:** Mirko Bakula, Tvrtko Hudolin, Tomislav Kulis, Toni Zekulic, Jerko Andelic, Zoran Zimak, Bojan Cikic, Ilija Juric, Zeljko Kastelan

**Affiliations:** ^1^ Department of Urology, University Hospital Centre Zagreb, Zagreb, Croatia; ^2^ School of Medicine, University of Zagreb, Zagreb, Croatia

**Keywords:** Bacillus Calmette-Guérin, mitomycin C, electromotive drug administration, non-muscle invasive bladder cancer, intravesical therapy

## Abstract

Intravesical therapy plays a crucial role in reducing the risk of recurrence and progression in patients with non-muscle invasive bladder cancer (NMIBC). Among the most widely used intravesical treatments is Bacillus Calmette-Guérin (BCG). To enhance therapeutic outcomes, sequential treatment strategies have been explored, including the combination of BCG with Mitomycin C (MMC) delivered via Electromotive Drug Administration (EMDA). In this retrospective clinical study, we report the results of sequential intravesical administration of BCG and MMC administered via EMDA (MMC EMDA) in 25 patients with intermediate- and high-risk NMIBC. Primary tumors were observed in 11 patients, while 14 had recurrent disease. Only one patient experienced recurrence during follow-up, after the 3 months of the therapy, resulting in an overall recurrence rate of 4%. The median follow-up duration was 16 months. In conclusion, our results support and expand the evidence indicating that sequential BCG and MMC EMDA offer a highly effective treatment approach for patients with intermediate- and high-risk NMIBC.

## 1 Introduction

Approximately 75% of individuals diagnosed with bladder cancer present with tumors confined to the mucosa (stage Ta, carcinoma *in situ*) or submucosa (stage T1) (1). These early-stage patients generally have better long-term survival, leading to a higher disease prevalence compared to those with more advanced stages (2). In this group, the prediction and prevention of recurrence and progression remains the primary clinical goal. To address this, risk stratification systems have been developed to guide treatment decisions (3).

An important subgroup within non–muscle-invasive bladder cancer (NMIBC) is patients with primary carcinoma *in situ* (CIS), where, if left untreated, approximately 54% will progress to muscle-invasive disease (4). While intravesical chemotherapy has not been shown to reduce progression, intravesical immunotherapy with Bacillus Calmette-Guérin (BCG) has demonstrated significant efficacy in reducing both recurrence and progression in intermediate- and high-risk NMIBC patients (5). When compared to intravesical chemotherapy using mitomycin C (MMC), BCG has been more effective in lowering recurrence rates (6). However, BCG therapy is also associated with a higher rate of side effects compared to chemotherapy, particularly with prolonged treatment or maintenance protocols (7). Although serious complications are rare, systemic BCG infection, including tuberculosis, occurs in approximately 1% of cases (8).

BCG and MMC act through distinct mechanisms—BCG activates the local immune response, while MMC exerts its effect by cross-linking DNA and inducing apoptosis. To optimize MMC’s efficacy, enhancing its penetration into bladder tissue is crucial. This can be achieved by increasing urothelial permeability through BCG-induced inflammation or by employing electromotive drug administration (EMDA), which facilitates active diffusion into cells at rates 4–7 times higher than passive diffusion (9). Sequential administration of BCG followed by MMC, especially when combined with EMDA, may provide a synergistic therapeutic benefit by improving drug delivery while balancing toxicity. In a randomized controlled trial using MMC with EMDA, sequential BCG plus MMC with EMDA improved recurrence-free survival and reduced progression compared to BCG monotherapy (10). However, in patients with pure CIS, the combination of BCG and MMC did not outperform BCG alone (11).

This study aimed to assess recurrence outcomes in intermediate- and high-risk NMIBC patients with or without concomitant CIS treated with a sequential intravesical BCG and MMC regimen at a single institution.

## 2 Patients and methods

### 2.1 Study design and population

This retrospective study included 25 patients diagnosed with intermediate- and high-risk NMIBC, with or without CIS, who received sequential intravesical therapy with BCG followed by MMC between August 2022 and April 2024. Risk stratification followed the criteria outlined by the European Urological Association (**Table 1**). Patients were included if they had complete data on treatment and follow-up.

**Table 1:** Patients.

Gender	N (%)
Male	18 (72)
Female	7 (28)
EAU risk group	N (%)
Very High	2 (8)
High	13 (52)
Intermediate	10 (40)
Recurrence	N (%)
Primary	14 (56)
Recurrent	11 (44)
Histology	N (%)
pTaHG + CIS	1 (4%)
pTaLG + CIS	2 (8%)
pT1HG + CIS	2 (8%)
CIS	1 (4%)
pTaLG	7 (28%)
pTaHG	3 (12%)
pT1HG	9 (36%)

### 2.2 Treatment protocol

All patients underwent TURBT before starting intravesical therapy. The treatment protocol included induction with 9 weekly instillations (BCG, BCG, MMC EMDA, BCG, BCG, MMC EMDA, BCG; BCG, MMC EMDA), followed by maintenance of 9 monthly installations (MMC EMDA, MMC EMDA, BCG, MMC EMDA, MMC EMDA, BCG, MMC EMDA, MMC EMDA, BCG) (9, 10, 12). Follow-up assessments were conducted every 3 months, involving office cystoscopy, cytologic examination of urine, blood tests and imaging studies. If suspicious cystoscopic or imaging findings, or positive cytologic results were observed during the follow-up, patients underwent additional necessary surgical procedures. Treatment was continued in patients who did not show signs of disease recurrence or showed low-grade recurrence, while it was discontinued in those with HG recurrence.

### 2.3 Data collection

Demographic data, tumor histology, EAU risk classification, treatment dates, and recurrence events were collected. Recurrence was defined as histologically confirmed tumor detected during follow-up. The side effects and missed treatment data were also collected.

### 2.4 Statistical analysis

Descriptive statistics were used to summarize patient characteristics. Recurrence rates were calculated, and Kaplan–Meier survival curves were constructed using recurrence as the event and follow-up time in months. Subgroup analyses were performed based on EAU risk classification and tumor type (primary vs. recurrent). Statistical analysis and data visualization were conducted using Microsoft Excel 2024 (Microsoft Corp., Redmond, WA, USA) and SPSS Statistics version 19.0 (IBM Corp., Armonk, NY, USA).

## 3 Results

**Figure 1:**
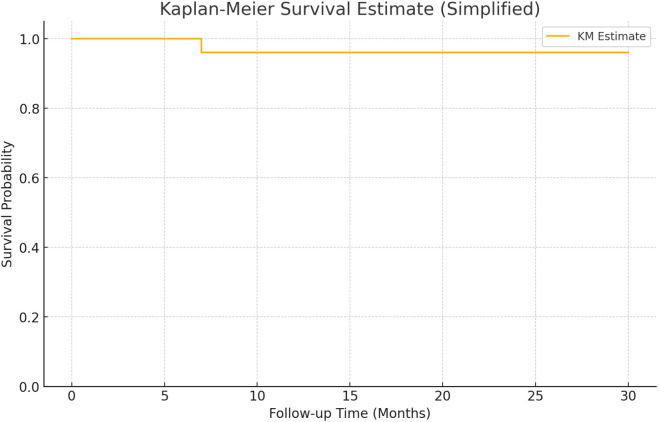
Survival estimate.

The cohort included 25 patients, 18 males and 7 females with a median age of 70 years. Most patients were classified as high-risk (13), followed by intermediate (10), and very high (2). Primary tumors were observed in 11 patients, while 14 had recurrent disease. Only one patient experienced recurrence during follow-up, after the 3 months of the therapy, resulting in an overall recurrence rate of 4%. This patient belonged to the intermediate-risk group and had a primary tumor. No recurrences occurred in high- or very high-risk groups. Kaplan-Meier analysis showed high survival probability across all groups, with a minor drop in the intermediate-risk group (**Figure 1**). The median follow-up duration was 16 months (range 7–30 months). In five patients, the intravesical installation protocol was not completed. The reasons for discontinuation were as follows: one patient experienced tumor recurrence during therapy; one patient required active treatment for concurrent prostate cancer; one patient developed persistent hematuria; and two patients discontinued due to loss of compliance. Additionally, one patient was diagnosed with genitourinary tuberculosis involving the prostate. This patient was also found to have synchronous lung cancer.

## 4 Discussion

Despite its established efficacy, BCG therapy for NMIBC faces several contemporary challenges. Recurrence remains a major concern, with 20–40% of patients experiencing disease relapse or progression to BCG-refractory states (13). Variability in reported outcomes across studies often reflects differences in patient populations, tumor characteristics, treatment regimens, and surveillance protocols. In addition, BCG is associated with considerable tolerability issues; up to 60–70% of patients experience local or systemic adverse effects which may lead to treatment discontinuation (14, 15). A further critical issue is the recent global shortage of BCG, which stems from both rising demand and limitations in the production and distribution infrastructure.

In response to the 2019 shortage, the American Urological Association (AUA) issued revised guidelines aimed at optimizing BCG allocation which included recommendations such as dose reduction, limiting maintenance therapy, prioritizing induction therapy for high-risk patients, and considering alternative therapies for intermediate-risk disease (16). In most cases of high-risk disease, radical cystectomy is obligated to be offered to eligible patients since it has remained the gold standard treatment in prevention of progression to life-threatening disease. However, because of its well-known significant morbidity and mortality as well as impact on quality of life afterwards, cystectomy is not an option favored by many patients.

Collectively, these efficacy, tolerability, quality of life and therapeutic accessibility concerns have driven the development and investigation of alternative intravesical therapies for NMIBC. Most prominent alternatives include chemotherapeutic agents such as valrubicin, mitomycin C, gemcitabine, and docetaxel, used alone or in sequential combinations (e.g., gemcitabine followed by docetaxel), which have shown promising efficacy and tolerability. Recently, newer immunotherapeutic agents like nadofaragene firadenovec (a gene therapy delivering interferon alfa-2b) and checkpoint inhibitors such as pembrolizumab have received FDA approval or are being investigated in trials, offering additional options for BCG-unresponsive disease. These developments reflect a growing trend toward personalized, mechanism-driven treatment approaches in NMIBC, aiming to optimize outcomes while minimizing toxicity (17).

One part of the spectrum of recent advances in the treatment of NMIBC has goal in optimizing the intravesical penetration of chemotherapy using device-assisted technology. The three most commonly used devices are: electromotive drug administration (EMDA) (18–20); radiofrequency-induced thermotherapy (RITE) (21); and hyperthermic intravesical chemotherapy (HIVEC) (22). Although, all mentioned device-assisted therapies have been published as a potential alternative to BCG, the most effective treatment schedule—whether standard or device-assisted—has yet to be established, and it remains unclear which types of patients are most likely to benefit from these approaches.

We have followed the usual application schedule introduced in the most accepted papers regarding this treatment, mostly by Di Stasi and colleagues (9, 10, 23). The rationale in combining immunotherapeutic BCG and chemotherapeutic MMC is based on the need to increase efficacy of these drugs and reduce emergence of resistant malignant cells: BCG-induced inflammation makes the bladder mucosa more permeable so that mitomycin reaches the target tissue more easily. The number of BCG doses is 6, the same as monotherapy. Therefore, the therapeutic regimen of 6 doses of BCG is maintained, but every two doses of BCG, MMC is administered with EMDA. It is proposed that induction of inflammation by use of 2 doses of BCG before MMC treatment makes the bladder mucosa more permeable and thus enables mitomycin to reach the target more easily and, as a result, the anti-tumor effect is enhanced. Therefore, the timing schedule and delivery techniques used in this protocol were selected to keep the benefits of both drugs at an optimum. The dosage of MMC is 40mg with intravesical electric current of 20 mA for 20 min. In the maintenance cycle, as seen in published data by Di Stasi et al, it was preferred to administer BCG after two administrations of mitomycin with EMDA because the immune response to BCG fades after about 6 months, a booster dose every 3 months is reasonable. Electromotive delivery exploits the potential of MMC to eliminate malignant cells that escape immunological surveillance induced by BCG. In the past, Rinthala and colleagues had already evaluated this monthly maintenance regimen in a ratio 1:2 of BCG to MMC (24).

In our study we demonstrated a notably low recurrence rate of 4% among patients with NMIBC treated with a sequential intravesical regimen of BCG followed by MMC administered via EMDA. Over a median follow-up period of 16 months, no cases of progression were observed, and recurrence occurred in only one patient—an intermediate-risk case with a primary tumor. These 5outcomes are particularly promising given the high proportion of patients with high- and very high-risk disease, which typically confers a higher likelihood of recurrence and progression despite therapy.

Our results align with earlier evidence supporting the efficacy of sequential BCG and MMC therapy, especially when drug delivery is enhanced through EMDA. Di Stasi et al. demonstrated that this combination significantly improved recurrence-free survival and reduced disease progression in patients with high-risk NMIBC, particularly in those who had failed prior BCG therapy, by leveraging the inflammatory effects of BCG to improve MMC penetration via EMDA (25). Similarly, Sanz Gomez et al. reported the results in 22 patients with HR NMIBC patients who had failed prior BCG treatment (26). The treatment regimen during induction and one year of maintenance achieved complete response rates of 95.5% at 3 months, 81% at 12 months, and 70% at 24 months. Only one patient progressed to muscle-invasive disease, and treatment was generally well tolerated, with mild adverse effects in 22% of patients. The study suggests this sequential approach may be a viable bladder-sparing option in selected BCG-refractory NMIBC patients.

In another study on patients with BCG failure, Juvet et al. evaluated the efficacy and safety in 26 patients, 14 of whom had been diagnosed with carcinoma *in situ*, 9 with T1, and 3 with Ta high-grade disease. Progression-free survival was approximately 58.3% at 1 year and 48.9% at 2 years, while recurrence-free survival was 41.9% at 1 year and 27.2% at 2 years. Notably, four patients (15.4%) died from bladder cancer by two years, highlighting the need for close surveillance despite some bladder-preserving benefit. The findings suggest that BCG/EMDA-MMC is a viable salvage option in BCG-unresponsive NMIBC, though long-term outcomes remain scarce (27).

While BCG-based therapies remain a cornerstone for NMIBC management, there is growing interest in chemotherapy-only alternatives—particularly for BCG-naïve patients or those who are unable to tolerate BCG. Among these, sequential intravesical gemcitabine and docetaxel (Gem/Doce) has shown promise. Compared to BCG + MMC, the Gem/Doce regimen offers a non-immunogenic approach that may be better suited for patients at higher risk of BCG-related complications, though long-term comparative data remain limited (28, 29).

Despite the encouraging outcomes in our study, 20% of patients discontinued therapy, including one due to persistent hematuria and another due to BCG-related genitourinary tuberculosis. Regarding toxicity, we used Common Terminology Criteria for Adverse Events (CTCAE) grading system that is a standardized system developed by the National Cancer Institute (NCI) to classify the severity of adverse events (AEs) experienced by patients in clinical trials (30). None of the patients experienced a severe systemic hypersensitivity reaction to MMC. One patient (4%) had an urticaria evolve during a transdermal EMDA procedure, caused by the electrical current itself (CTCAE grade 1, Skin and subcutaneous tissue disorders, Urticaria). Since the catheter used for MMC instillation is equipped with a small internal metal plate, it is more rigid than a standard Foley catheter. Therefore, urinary catheter placement requires more experience and skill to avoid urethral injury. In our institution, catheter insertion is performed by a qualified physician. Nevertheless, in two patients (8%) mild hematuria occurred on one occasion, which led to postponement of intravesical therapy for one week (CTCAE grade 1, Renal and urinary disorders, Hematuria). In one patient, persistent mild hematuria led to discontinuation of the therapy (CTCAE grade 1, Renal and urinary disorders, Hematuria). With respect to LUTS after MMC EMDA treatment, mild post-treatment urinary frequency (CTCAE grade 1, Renal and urinary disorders, Urinary frequency) occurred at least once during the therapy in 8 patients (32%) and regressed within 24 hours. Specific treatment was not necessary.

This study’s limitations include its small sample size, retrospective design, relatively short follow-up, and the lack of a control group. Larger prospective studies are needed to validate the efficacy of BCG + MMC + EMDA regimens and to compare them directly with emerging alternatives such as Gem/Doce. Additionally, future research should investigate biomarkers predictive of response and explore optimal sequencing strategies for immunotherapy and chemotherapy combinations in NMIBC.

A notable strength of this study is its contribution to a field where evidence remains limited, as data on the use of MMC EMDA in combination with BCG therapy are scarce. The study also offers clinically meaningful insights, with findings that demonstrate good applicability in routine practice. Moreover, the consistency of our results with previously published studies further supports the robustness and external validity of the observed outcomes.

In summary, our findings add to a growing body of literature suggesting that sequential BCG and MMC therapy enhanced with EMDA is a promising option for patients with intermediate- and high-risk NMIBC. Interestingly, the recurrence was observed in patient with primary tumor, which could suggest that newly diagnosed NMIBC may behave differently than recurrent disease in response to sequential therapy—a hypothesis warranting further investigation. Emerging chemotherapy-only regimens such as Gem/Doce further enrich the therapeutic landscape and highlight the need for flexible, evidence-based treatment paradigms tailored to patient risk profiles and tolerability.

Non-muscle invasive bladder cancer; Bacillus Calmette-Guérin; Mitomycin C; Electromotive Drug Administration; Intravesical therapy.

## Data Availability

The raw data supporting the conclusions of this article will be made available by the authors, without undue reservation.

## References

[1] Compérat E Larré S Roupret M Neuzillet Y Pignot G Quintens H. Clinicopathological characteristics of urothelial bladder cancer in patients less than 40 years old. Virchows Arch Int J Pathol. (2015) 466:589–94. doi: 10.1007/s00428-015-1739-2, PMID: 25697540

[2] Burger M Catto JWF Dalbagni G Grossman HB Herr H Karakiewicz P. Epidemiology and risk factors of urothelial bladder cancer. Eur Urol. (2013) 63:234–41. doi: 10.1016/j.eururo.2012.07.033, PMID: 22877502

[3] Sylvester RJ Rodríguez O Hernández V Turturica D Bauerová L Bruins HM. European association of urology (EAU) prognostic factor risk groups for non-muscle-invasive bladder cancer (NMIBC) incorporating the WHO 2004/2016 and WHO 1973 classification systems for grade: an update from the EAU NMIBC guidelines panel. Eur Urol. (2021) 79:480–8. doi: 10.1016/j.eururo.2020.12.033, PMID: 33419683

[4] Lamm DL. Carcinoma in *situ* . Urol Clin North Am. (1992) 19:499–508. 1636234

[5] Shelley MD Kynaston H Court J Wilt TJ Coles B Burgon K. A systematic review of intravesical bacillus Calmette-Guérin plus transurethral resection vs transurethral resection alone in Ta and T1 bladder cancer. BJU Int. (2001) 88:209–16. doi: 10.1046/j.1464-410x.2001.02306.x, PMID: 11488731

[6] Schmidt S Kunath F Coles B Draeger DL Krabbe LM Dersch R. Intravesical Bacillus Calmette-Guérin versus mitomycin C for Ta and T1 bladder cancer. Cochrane Database Syst Rev. (2020) 1:CD011935. doi: 10.1002/14651858.CD011935.pub2, PMID: 31912907 PMC6956215

[7] Sylvester RJ van der MEIJDEN APM Lamm DL. Intravesical bacillus Calmette-Guerin reduces the risk of progression in patients with superficial bladder cancer: a meta-analysis of the published results of randomized clinical trials. J Urol. (2002) 168:1964–70. doi: 10.1016/S0022-5347(05)64273-5, PMID: 12394686

[8] Larsen ES Nordholm AC Lillebaek T Holden IK Johansen IS. The epidemiology of bacille Calmette-Guérin infections after bladder instillation from 2002 through 2017: a nationwide retrospective cohort study. BJU Int. (2019) 124:910–6. doi: 10.1111/bju.14793, PMID: 31054198

[9] Di Stasi SM Vespasiani G Giannantoni A Massoud R Dolci S Micali F. Electromotive delivery of mitomycin C into human bladder wall. Cancer Res. (1997) 57:875–80. 9041189

[10] Di Stasi SM Giannantoni A Giurioli A Valenti M Zampa G Storti L. Sequential BCG and electromotive mitomycin versus BCG alone for high-risk superficial bladder cancer: a randomised controlled trial. Lancet Oncol. (2006) 7:43–51. doi: 10.1016/S1470-2045(05)70472-1, PMID: 16389183

[11] Kaasinen E Wijkström H Rintala E Mestad O Jahnson S Malmström PU. Seventeen-year follow-up of the prospective randomized Nordic CIS study: BCG monotherapy versus alternating therapy with mitomycin C and BCG in patients with carcinoma in *situ* of the urinary bladder. Scand J Urol. (2016) 50:360–8. doi: 10.1080/21681805.2016.1210672, PMID: 27603424

[12] Hayne D Stockler M McCombie SP Chalasani V Long A Martin A. BCG+MMC trial: adding mitomycin C to BCG as adjuvant intravesical therapy for high-risk, non-muscle-invasive bladder cancer: a randomised phase III trial (ANZUP 1301). BMC Cancer. (2015) 15:432. doi: 10.1186/s12885-015-1431-6, PMID: 26014129 PMC4445809

[13] Lidagoster S Ben-David R De Leon B Sfakianos JP. BCG and alternative therapies to BCG therapy for non-muscle-invasive bladder cancer. Curr Oncol. (2024) 31:1063–78. doi: 10.3390/curroncol31020079, PMID: 38392073 PMC10888316

[14] Fuge O Vasdev N Allchorne P Green JS. Immunotherapy for bladder cancer. Res Rep Urol. (2015) 7:65–79. doi: 10.2147/RRU.S63447, PMID: 26000263 PMC4427258

[15] Sfakianos JP Kim PH Hakimi AA Herr HW. The effect of restaging transurethral resection on recurrence and progression rates in patients with nonmuscle invasive bladder cancer treated with intravesical bacillus Calmette-Guérin. J Urol. (2014) 191:341–5. doi: 10.1016/j.juro.2013.08.022, PMID: 23973518 PMC4157345

[16] American Urological Association. Available online at: https://www.auanet.org/bcg-shortage-notice (Accessed July 01, 2025).

[17] Li Y Youssef SF Buanz AB. Intravesical combination therapies for non-muscle invasive bladder cancer: Recent advances and future directions. Eur J Pharmacol. (2022) 926:175024. doi: 10.1016/j.ejphar.2022.175024, PMID: 35580708

[18] Di Stasi SM Riedl C. Updates in intravesical electromotive drug administration of mitomycin-C for non-muscle invasive bladder cancer. World J Urol. (2009) 27:325–30. doi: 10.1007/s00345-009-0389-x, PMID: 19234707

[19] Racioppi M Di GianFrancesco L Ragonese M Palermo G Sacco E Bassi PF. ElectroMotive drug administration (EMDA) of Mitomycin C as first-line salvage therapy in high risk “BCG failure” non muscle invasive bladder cancer: 3 years follow-up outcomes. BMC Cancer. (2018) 18:1224. doi: 10.1186/s12885-018-5134-7, PMID: 30522445 PMC6282335

[20] Melgarejo-Segura MT Morales-Martínez A Yáñez-Castillo Y Arrabal-Polo MÁ Gómez-Lechuga P Pareja-Vílchez M. A systematic review of the efficacy of intravesical electromotive drug administration therapy for non-muscle invasive bladder cancer. Urol Oncol. (2023) 41:166–76. doi: 10.1016/j.urolonc.2022.09.016, PMID: 36328923

[21] Colombo R van Valenberg H Moschini M Witjes JA. Radiofrequency-induced thermo-chemotherapy effect (RITE) for non muscle invasive bladder cancer treatment: current role and perspectives. Urologia. (2016) 83:7–17. doi: 10.5301/uro.5000197, PMID: 27768213

[22] Angulo JC Álvarez-Ossorio JL Domínguez-Escrig JL Moyano JL Sousa A Fernández JM. Hyperthermic mitomycin C in intermediate-risk non-muscle-invasive bladder cancer: results of the HIVEC-1 trial. Eur Urol Oncol. (2023) 6:58–66. doi: 10.1016/j.euo.2022.10.008, PMID: 36435738

[23] Di Stasi SM Giannantoni A Massoud R Dolci S Navarra P Vespasiani G. Electromotive versus passive diffusion of mitomycin C into human bladder wall: concentration-depth profiles studies. Cancer Res. (1999) 59:4912–8., PMID: 10519404

[24] Rintala E Jauhiainen K Rajala P Ruutu M Kaasinen E Alfthan O. Alternating mitomycin C and bacillus Calmette-Guerin instillation therapy for carcinoma in *situ* of the bladder. Finnbladder Group J Urol. (1995) 154:2050–3. 7500456

[25] Di Stasi SM Valenti M Verri C Liberati E Giurioli A Leprini G. Electromotive instillation of mitomycin immediately before transurethral resection for patients with primary urothelial non-muscle invasive bladder cancer: a randomised controlled trial. Lancet Oncol. (2011) 12:871–9. doi: 10.1016/S1470-2045(11)70190-5, PMID: 21831711

[26] Sanz Gómez I Huguet J Bravo A Robalino J Rodríguez Faba Ó Territo Á. Sequential treatment with bacillus calmette-güerin (BCG) and mitomycin C administered with electromotive drug administration (EMDA) in patients with high-risk nonmuscle invasive bladder cancer after BCG failure. Clin Genitourin Cancer. (2023) 21:e286–90. doi: 10.1016/j.clgc.2023.03.002, PMID: 37076337

[27] Juvet T Mari A Lajkosz K Wallis CJ Kuk C Erlich A. Sequential administration of Bacillus Calmette-Guerin (BCG) and Electromotive Drug Administration (EMDA) of mitomycin C (MMC) for the treatment of high-grade nonmuscle invasive bladder cancer after BCG failure. Urol Oncol. (2020) 38:850.e9–850.e15. doi: 10.1016/j.urolonc.2020.06.031, PMID: 32712139

[28] McElree IM Steinberg RL Mott SL O’Donnell MA Packiam VT. Comparison of sequential intravesical gemcitabine and docetaxel vs bacillus calmette-guérin for the treatment of patients with high-risk non-muscle-invasive bladder cancer. JAMA Netw Open. (2023) 6:e230849. doi: 10.1001/jamanetworkopen.2023.0849, PMID: 36853609 PMC9975907

[29] Bakula M Hudolin T Knezevic N Zimak Z Andelic J Juric I. Intravesical gemcitabine and docetaxel therapy for BCG-naïve patients: A promising approach to non-muscle invasive bladder cancer. Life (Basel). (2024) 14:789. doi: 10.3390/life14070789, PMID: 39063544 PMC11278229

[30] U.S. Department of Health and Human Services, National Institutes of Health, National Cancer Institute. Common Terminology Criteria for Adverse Events (CTCAE), version 5.0. (2017). Available online at: https://dctd.cancer.gov/research/ctep-trials/for-sites/adverse-events (Accessed July 01, 2025).

